# Glypican-1 and glycoprotein 2 bearing extracellular vesicles do not discern pancreatic cancer from benign pancreatic diseases

**DOI:** 10.18632/oncotarget.26620

**Published:** 2019-02-01

**Authors:** Fabrice Lucien, Vivian Lac, Daniel D. Billadeau, Ayelet Borgida, Steven Gallinger, Hon S. Leong

**Affiliations:** ^1^ Department of Urology, Mayo Clinic, Rochester, MN, USA; ^2^ Department of Pathology and Laboratory Medicine, Western University, London, ON, Canada; ^3^ Department of Oncology, Mayo Clinic, Rochester, MN, USA; ^4^ Zane Cohen Centre for Digestive Diseases, Mount Sinai Hospital, Toronto, ON, Canada; ^5^ Department of Surgery, University Health Network, Toronto, ON, Canada

**Keywords:** pancreatic cancer, flow cytometry, extracellular vesicles, glypican-1, liquid biopsy

## Abstract

Pancreatic ductal adenocarcinoma (PDAC) is a lethal disease that is clinically asymptomatic in its early stages of development. Non-invasive testing for pancreatic cancer biomarkers would significantly improve early detection and patient care. Extracellular vesicles (EVs) are circulating tumor fragments present in the blood and may express cancer specific biomarkers that would enable early detection of pancreatic cancer. We tested the utility of a blood test enumerating EVs positive for the pancreas-specific marker Glycoprotein 2 (GP2) and the putative pancreatic cancer marker Glypican-1 (GPC1) in patients with PDAC. Various levels of GPC1-positive and GP2/GPC1-positive EVs were detected in PDAC patients but were not significantly higher than benign pancreatic disease (BPD) patients. The sensitivity and specificity of the GPC1 EV test was 26.67% and 87.50% respectively, whereas the sensitivity and specificity for the GPC1+GP2 EV test was 23.33% and 90.00% respectively. Immunohistochemistry of GPC1 expression in a tissue microarray of PDAC and various controls also did not demonstrate specificity of GPC1 to PDAC. Hence, enumeration of GPC1-positive EVs, solely or in conjunction with GP2, was unable to effectively distinguish between BPD and pancreatic cancer.

## INTRODUCTION

Pancreatic cancer is lethal and is the fourth leading cause of cancer-related deaths in North America [1, 2]. From diagnosis, the five-year survival rate of pancreatic cancer is approximately 5%, with most patients dying within several months after diagnosis [1]. The early stages of pancreatic cancer are largely asymptomatic or clinically silent with symptoms only appearing once the cancer has invaded neighboring tissues or has metastasized to distant sites [3]. At this point, therapeutic intervention is palliative and therefore early detection of this disease is critical. A non-invasive blood test for the early detection of pancreatic cancer would be immensely beneficial but must have excellent performance test characteristics because of its low prevalence in the general population. While there is a paucity of biomarkers presumed specific for pancreatic cancer, these are often unable to discern pancreatic cancer from other cancers and/or benign pancreatic diseases (BPD), including pancreatitis and pancreatic cysts [4–7]. The most well-characterized pancreatic cancer biomarker, Carbohydrate Antigen 19-9 (CA19-9), only has a positive predictive value of 0.5–0.9% when used in the screening of asymptomatic individuals [8].

Next generation “liquid biopsies” for pancreatic cancer have emerged and have largely focused on the detection of circulating tumor DNA (ctDNA), circulating tumor cells (CTCs) or tumor-derived extracellular vesicles. Such liquid biopsies represent an attractive and non-invasive means for monitoring cancer progression and molecular changes within tumor cells within patients [9, 10]. However promising, caveats remain in the clinical validation for these blood tests. For instance, CTCs exist in extremely low concentrations of just one cell among many millions of blood cells [9]. This may be even lower in patients with early-stage cancers that have not yet metastasized, wherein tumor cells have little to no ability to intravasate into the vasculature [10]. Circulating tumor DNA (ctDNA) has a low half-life and may not be present at high enough levels for analysis. On the other hand, EVs, which are small, membrane-bound particles released in abundance by most cells of the body into the vasculature [11–15], have been shown to be released by tumor cells at an increasing rate with cancer progression [16, 17]. These EVs contain a variety of cargo from their originating cells including protein, DNA, and RNA [18, 19] which may provide valuable information on any tumor cells they are released from. Therefore, an effective liquid biopsy for cancer detection may require enumeration of tumor-derived EVs such as exosomes.

A recent study by *Melo et al*. demonstrated the clinical utility of Glypican-1 (GPC1) on pancreatic cancer exosomes and the levels of Glypican 1 expressing exosomes (GPC1+ve exosomes) to be extremely capable of identifying early- and late-stage pancreatic cancer from healthy individuals or patients with BPD (AUC=1.0) [20]. Since these GPC1+ve exosomes were observed to be 100-175nm in diameter [20], it is conceivable that other EVs such as microparticles/microvesicles released by pancreatic cancer cells could bear the GPC1 biomarker. GPC1 is a pan-specific marker for cancer that is not only elevated in pancreatic cancer [20, 21], but is also elevated in other neoplastic diseases, such as breast cancer and gliomas [22, 23]. To validate the use of GPC1+ve EVs for screening and detection of pancreatic cancer, we utilized nanoscale flow cytometry which is an instrument specialized for high-throughput and multi-parametric analysis of Evs [24, 25]. Nanoscale flow cytometry is capable of identifying EVs between 100-1,000nm in diameter, and is equipped with lasers and filters used in conventional flow cytometry to detect any desired combination of surface biomarkers on Evs [26]. This gating strategy would allow us to determine the clinical utility of all GPC1+ve EVs, whether they were exosomes, microvesicles/microparticles in identifying patients with pancreatic cancer from patients with benign conditions. We also sought to combine GPC1 analysis with an additional pancreatic tissue specific marker, Glycoprotein-2 (GP2), a major membrane protein specific for secretory granules of the exocrine pancreas [27, 28]. GP2 has been detected in the blood of patients affected by various pancreatic diseases including both pancreatic cancer and pancreatitis [29], thus we sought to enumerate GPC1-GP2 dual positive EVs in patient plasma samples representing various stages of pancreatic cancer development.

## RESULTS

### Circulating GPC1^+^GP2^+^-positive EVs are detected in human plasmas by nanoscale flow cytometry

**Figure 1:**
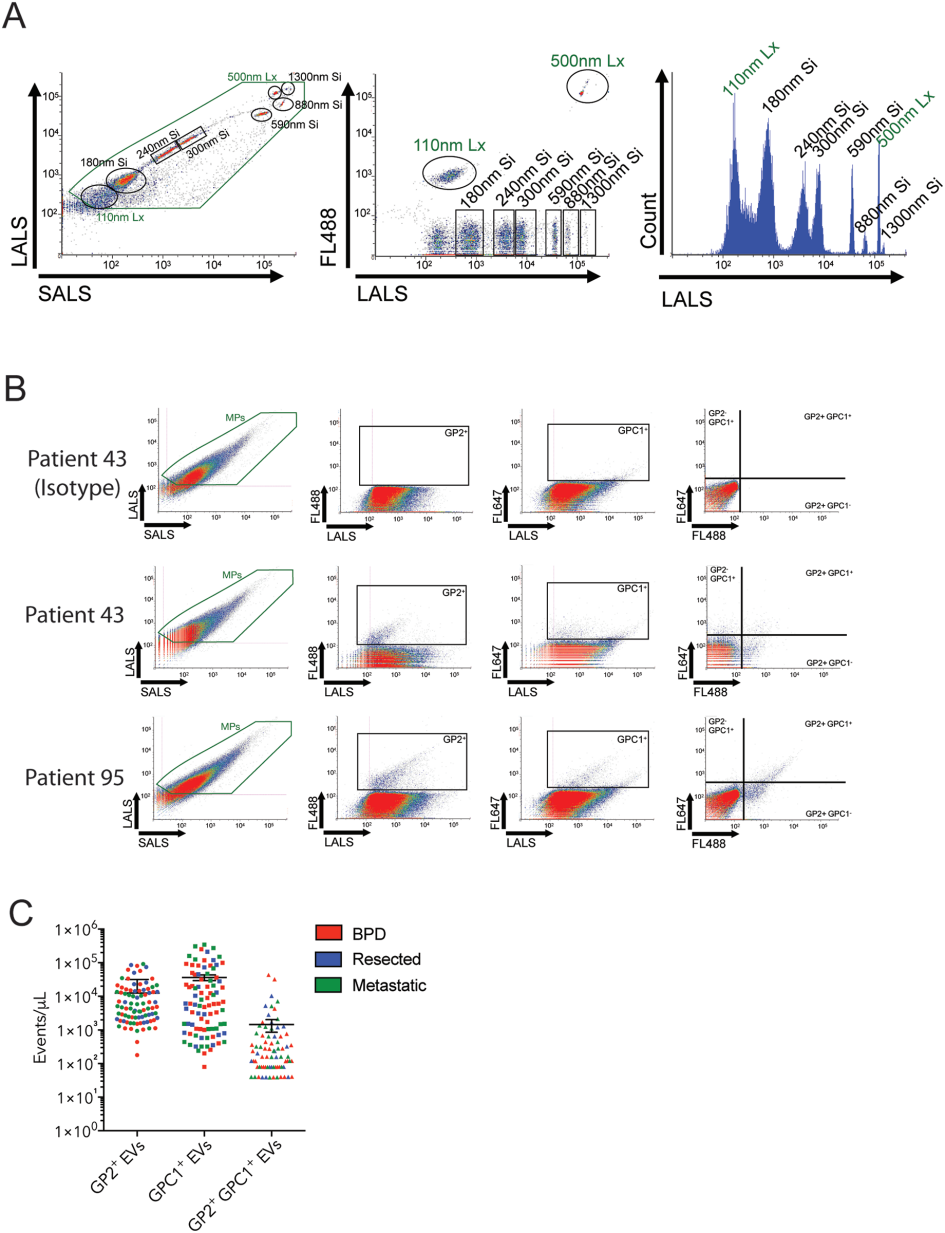
Circulating GP2+ GPC1+ EVs are detected in human plasmas by nanoscale flow cytometry **(A)** Cytograms and histograms showing calibration bead distribution with light scatter (LALS, SALS) and fluorescence detection (FL488). The green box indicates microparticle-related events (110-1000nm). **(B)** Scatterplots representing the size distribution, GP2+ve events, GPC1+ve events and double positive events from plasma of two different PDAC patients representing low (patient 43) and high levels (patient 95) of double positive EVs. **(C)** Graphs showing distribution of EV concentration for single positive (GP2 or GPC1) and double positive EVs in patient plasmas (n= 93 patients). Bars representing mean +/- s.e.m.

The Apogee A50-Micro nanoscale flow cytometer is capable of detecting and enumerating extracellular vesicles within a size range of 100-1,300 nm based on calibration beads (Figure 1A). To develop a GPC1 and GPC1+GP2 EV-based liquid biopsy, we optimized the incubation conditions for anti-GPC1 and anti-GP2 antibodies (Supplementary Figure 1). The addition and incubation of these antibodies to patient plasma samples prior to analysis using the A50-Micro nanoscale flow cytometer revealed the enrichment of subpopulations of GPC1- and GP2-positive EVs in some patient plasmas among all plasma EVs ranging from 100-1000 nm in diameter (Figure 1B-1C). A proportion of patients with moderate to high levels of GPC1- and GP2-positive EVs also revealed the enrichment of a GPC1+GP2-positve (dual-positive) subpopulation (Figure 1C); however, there were also many patients that showed elevated GPC1 and GP2 EVs with little to no enrichment of GPC1+GP2 EVs.

### Enumeration of GPC1^+^ EVs does not distinguish PDAC from BPD patients

**Figure 2:**
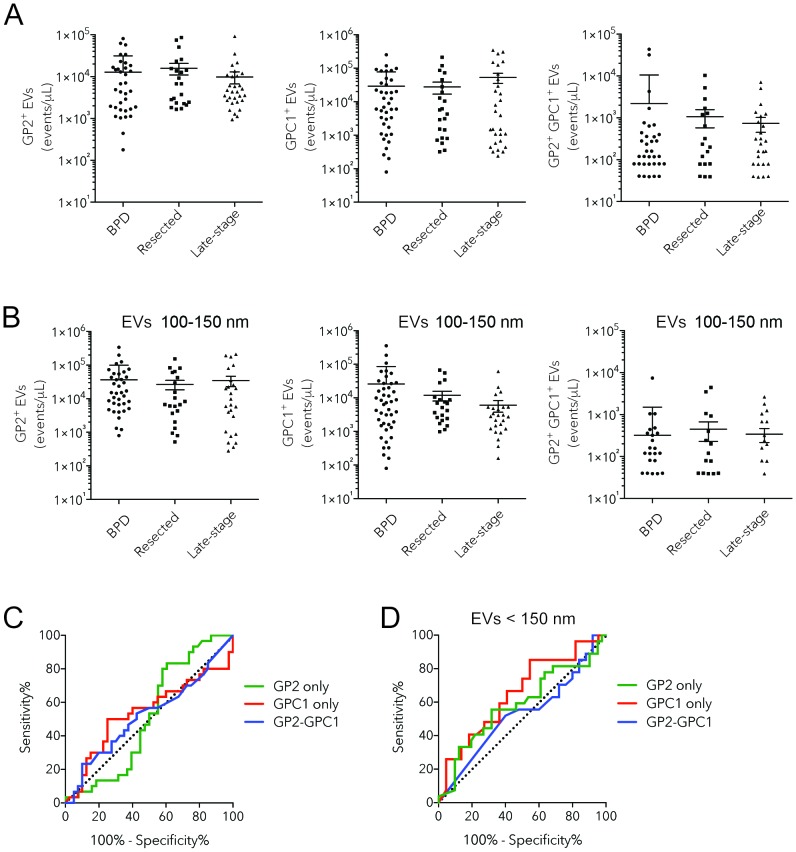
Assessment of levels of circulating GP2+ GPC1+ EVs as a biomarker for pancreatic cancer **(A-B)** Histograms showing enumeration of GP2, GPC1, and GP2 GPC1 EVs in plasmas of BPD (n= 40), resected pancreatic cancer (n= 23) and PDAC (n= 30) patients: all EVs (A), EVs smaller than 150 nm (B). **(C-D)** ROC curves of GPC1+ EV and GP2 GPC1 EV levels for distinguishing PDAC subjects from BPD patients: all EVs (C), EVs between 100-150 nm (D). Bars representing mean +/- s.e.m.

In a blinded study, we characterized the GP2+ve EV, GPC1+ve EV and GPC1-GP2+ve EV counts for all 93 patient plasma samples, correcting for the non-specific binding observed in corresponding isotype controls (Figure 2A, Supplementary Figure 1). GP2+ve, GPC1+ve and GPC1-GP2+ve EV counts were highly variable within the three different patient groups, from the total absence of positive EVs to tens or hundreds of thousands of positive EVs per microliter (Figure 2A). The majority of patients exhibited low levels of both GPC1+ve and GPC1-GP2+ve EVs, which is reflected by the lack of significant difference between mean EV counts for BPD, resected cancer, and metastatic cancer groups as determined through one-way ANOVA (p>0.20) (Figure 2A). Only 5 of 23 resected cancer patients and 9 of 30 metastatic cancer patients had mean GPC1 EV counts higher than the mean counts observed in BPD patients. This drops to 3 of 23 and 2 of 30 respectively when using GPC1-GP2 EV counts.

We looked at levels of GP2+ve EV, GPC1+ve EV and GPC1-GP2+ve EVs (Figure 2B). Although no significant differences were observed between patient groups for the three signatures, levels of GPC1+ve EVs were actually decreased in metastatic pancreatic cancer patients (6,062 EVs/microliter) compared to resected cancer (11,958 EVs/microliter) and BPD (25,911 EVs/microliter) groups.

To determine the performance test characteristics of the GPC1 and GPC1+GP2 EV tests for pancreatic cancer diagnosis, an ROC curve was generated (Figure 2B), which illustrates the trade-off of a diagnostic test between sensitivity and specificity. The AUC of the GPC1 EV and GPC1+GP2 EV test was 0.5404 and 0.5229 respectively (Table 2). The GPC1 EV test had a sensitivity and specificity of 26.67% and 87.5% respectively, at a cut-off of less than 604 GPC1-positive EVs per microliter. The best cut-off for the diagnosis of pancreatic cancer using the GPC1+GP2 EV test was greater than 780 EVs per microliter, yielding a sensitivity and specificity of 23.33% and 90.0% respectively (Table 2). If the EV test excludes all EVs larger than 150 nm, the GPC1 EV test has an AUC of 0.6524 and at a cut-off of less than 970 EVs per microliter, sensitivity and specificity were 25.93% and 95.45% respectively (Figure 2D, Table 3). It is important to note that these values cannot be directly compared with the sensitivity and specificity for pancreatic cancer biomarkers assessed in other studies since the negative outcome for the ROC curve analysis were BPD patients that were already shown to be falsely positive for pancreatic cancer using the CA 19-9 serum test. Therefore, these BPD patients represent outliers within the population of individuals that are not affected by pancreatic cancer.

**Table 1:** Patient Characteristics

		Pancreatic Cancer		Benign Pancreatic Diseases	
		Number of participants	% of participants	Number of participants	% of participants
		52		41	
Sex	Men	22	42.3%	9	21.9%
	Women	30	57.7%	32	78.1%
Median Age (range)		61 (21-88)		54 (21-73)	
AJCC stage	I	5	9.80%		
	II	0	0%		
	IIA	4	7.80%		
	IIB	13	25%		
	III	0	0%		
	IV	30	58.90%		
Tumor Resected	Yes	25	48.10%		
	No	27	51.90%		

**Table 2:** ROC parameters and 95% confidence intervals for GPC1 and GP2 EV Counts

Parameter	AUC	95% CI	Cut-off value	Sensitivity %	95% CI	Specificity%	95% CI
GP2-positive EVs (EVs/uL)	0.5145	0.3746-0.6543	>10200.0	26.67	12.28-45.89	60.53	43.39-75.96
GPC1-positive EVs (EVs/uL)	0.5404	0.3962-0.6846	<604.0	26.67	12.28-45.89	87.50	73.80-95.92
GPC1+GP2-positive EVs (EVs/uL)	0.5229	0.3824-0.6634	>780.0	23.33	9.934-42.28	90.00	76.34-97.21

**Table 3:** ROC curve parameters and 95% confidence intervals for GPC1 and GP2+GPC1 EV counts (EVs smaller than 150 nm only)

Parameter	AUC	95% CI	Cut-off value	Sensitivity %	95% CI	Specificity%	95% CI
GP2-positive EVs (EVs/uL)	0.5845	0.4405-0.7285	>10200.0	25.93	11.11-46.28	90.24	76.87-97.28
GPC1-positive EVs (EVs/uL)	0.6524	0.4972-0.8076	<972.0	25.93	11.11-46.28	95.45	77.16-99.88
GPC1+GP2-positive EVs (EVs/uL)	0.5237	0.3645-0.6829	<100.0	62.96	42.37-80.60	32.00	14.95-53.50

### GPC1 is weakly expressed in human PDAC

**Figure 3:**
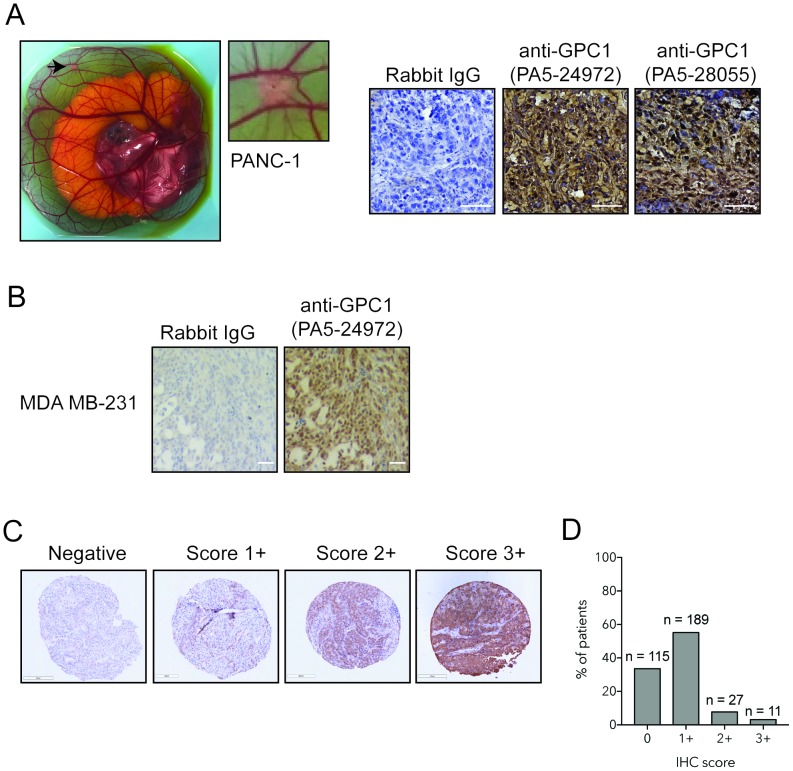
GPC1 expression in tissue microarray of human PDAC **(A)** Photomicrograph representing a PANC-1 tumor xenograft onplanted into chorioallantoic membrane of a chicken embryo. Immunohistochemical staining was performed with GPC1 antibody clones PA5-29472 PA5-28055. Bar: 20 μm. **(B)** Immunohistochemical staining of MDA-MB 231 cells with GPC1 antibody PA5-24972 or isotype-matched control (rabbit IgG), Bar: 20 μm. **(C)** Tissue Microarray of human PDAC tumors stained with GPC1 antibody PA5-29472. Representative images of differential expression of GPC1: negative (0), weak (1+), moderate (2+), strong (3+), Magnification 10x. **(D)** Immunohistochemical score for GPC1 expression in tissue microarray of human PDAC. N indicates number of cores for each IHC score.

We assessed GPC1 expression in a cohort of 140 different individuals presenting PDAC. We optimized immunohistochemical staining of GPC1 on pancreatic (PANC-1, Supplementary Figure 2) and breast cancer cells (MDA MB-231) obtained from tumor xenografts (Figure 3A-3B). Both antibodies raised against GPC1 showed strong positive staining in PANC-1 and cell surface staining was observed with GPC1 clone PA5-24972 (Figure 3A). Immunohistochemistry of GPC1 in the tissue microarray denotes a cytoplasmic and nuclear stain distribution (Figure 3C). GPC1 is mostly present in cytoplasm or at cell surface. By using a nuclear exclusion algorithm, the TMA indicated that 65% of cores were positive for cytoplasmic/surface GPC1 (Figure 3D). The majority of positive cores (55%) revealed a weak expression of GPC1 whereas moderate and strong expression was detected in 8% and 3% of cases respectively (Figure 3C-3D).

## DISCUSSION

In the present study, we have developed and optimized GPC1 and GPC1-GP2 dual positive EV-based liquid biopsies using nanoscale flow cytometry. These tests were capable of detecting elevated levels of GPC1-positive and GPC1-GP2 dual-positive EVs in a subset of individuals (Figure 1C, Figure 2A-2B). However, these EV levels were not specific to patients with pancreatic cancer. Overall, the presence of GPC1 fails to distinguish between patients with BPD and pancreatic cancer. BPD patients exhibited high levels of GPC1 and dual positive EVs. Most importantly, patients with PDAC did not all express high levels of GPC positive EVs or double positive EVs. A gating of only EVs between 110-150nm meant to enumerate GPC1+ve, GP2+ve or GPC2 and GP2 dual positive exosomes (small EVs) also failed to distinguish between patients with BPD and pancreatic cancer.

Whether through the analysis of GPC1 alone or in conjunction with GP2, target EV counts between all three groups did not significantly differ (Figure 2A-2B) and thus was not informative regarding pancreatic cancer status. Surprisingly, GPC1 was found to be enriched in a number of BPD patients and yet was found to be seldom present in the majority of patients with metastatic pancreatic cancer (Figure 2A). Combined analysis with GP2 was intended to improve the effectiveness of our EV-based test, however an even lower number of metastatic cancer patients exhibited elevated GPC1-GP2 EV counts compared to mean counts in BPD patients than GPC1 EV counts (2 of 30 patients compared to 9 of 30 patients) (Figure 2A). The rarity of GPC1-positive EVs and lack of consistent co-occurrence with GP2 in pancreatic cancer patients challenge the notion that GPC1 present on EVs is exclusively found and derived from cancer of the pancreas (given that patients are unaffected by other cancers).

The ROC curve further demonstrates the ineffectiveness of GPC1 EVs and GPC1+GP2 EVs in discerning BPD from pancreatic cancer (Figure 3). In this plot, both GPC1 and GPC1+GP2 EV tests closely follow the diagonal line, which represents a diagnostic test that only has a 50-50 chance at the correct diagnosis of a patient (Figure 2C). In fact, the best cut-off value for the GPC1 EV test was the diagnosis of pancreatic cancer if individuals present with less than 604 EVs per microliter when in fact EV counts should rise with cancer due to the increased abundance of cells expressing the biomarkers, thus revealing a lack of association of GPC1 EVs with pancreatic cancer. Despite the high specificity (90%) of GPC1+GP2 EVs at a cut-off of above 780 EVs per microliter, this test suffered from low sensitivity (23.33%) (Figure 2C, Table 2). Overall, the findings of this study led us to conclude that since GPC1 cannot discern late-stage, metastatic pancreatic cancer from benign pancreatic diseases, GPC1 is not useful in the early detection of pancreatic cancer.

The results of the tissue microarray analysis corroborated our “liquid biopsy” GPC1-EV results, with 55% of metastatic PDAC lesions expressing low levels of GPC1 via immunohistochemistry. These results are more in keeping with the results of TCGA and Protein Atlas in which high-throughput analyses independent of any antibody were used. This discrepancy emphasized the need for external validation work to be performed and presented in the same study.

The primary difference in our studies compared to other papers is that we utilized a high resolution flow cytometry instrument (nanoscale flow cytometry, nFC) that is designed to enumerate EVs whereas various other papers utilized independent methods such as mass spectrometry or ELISA [33, 34]. The use of nanoscale flow cytometry is a promising technique for EV enumeration and is superior than conventional flow cytometry instruments and FACS instruments in the analysis of EVs [25, 35]. Results observed with the use of conventional flow cytometry for EV analysis should be consistent when using nFC due to its increased sensitivity for measuring only EVs and not cells. The size restriction of our nanoscale flow cytometer limited the scope of our study to the analysis of EVs ranging from 100 to 1,000 nm in diameter. Smaller EVs often called “exosomes”, are detectable through our technology if exosomes larger than 100 nm fall under this definition. However, exosomes are primarily formed by the inward budding of endosomes and thus reflect a different cellular origin from larger EVs of 100-1,000 nm in diameter, often called “microparticles/microvesicles”, which are formed by the outward budding of the plasma membrane [11]. Since GPC1 is a membrane marker, it is likely that microparticles/microvesicles are the proposed GPC+ve EV and not exosomes but considering the broad definition and its inclusion criteria, nFC was used which will analyze both exosomes and microparticles/microvesicles.

A recent study by *Bailey et al*. found pancreatic cancer (specifically pancreatic ductal adenocarcinomas, which constitute the large majority of exocrine cancers) to consist of four distinct molecular subtypes [36]. These subtypes differ in the involvement of particular pathways as well as histopathological characteristics [36]. This suggests that pancreatic cancer is far more complicated than previously believed and it may not be possible to find a single biomarker capable of the early detection of all of these pancreatic cancer subtypes. The effectiveness of biomarkers pertaining to these specific subtypes may have gone unnoticed in this study or prior studies and thus the subtyping of pancreatic cancer patients should be characterized in future biomarker studies. The concept of a panel of EV biomarkers as opposed to one has already shown promise for diagnosis of pancreatic cancer such as Yang et al., in which five EV based biomarkers exhibited a significantly higher accuracy rate (94-100%) compared to GPC1 expressing EVs (56%) [37] might be more effective, especially given that a test with any false positive rate would generate concern when used across a large screening population. Machine learning to identify and build an algorithm based test from multi-parametric data such as EV panels [37] would also lead to a greater likelihood that a highly accurate test for pancreatic cancer be available in the near future [38].

In closing, nanoscale flow cytometry offers a rapid, quantitative, and multi-parametric approach to blood testing and putative “liquid biopsies” for other disease sites should use nFC for external validation. Developing a single biomarker to identify all pancreatic cancer subtypes is ideal but challenging, and specificity will likely arise from a unique combination of biomarkers present on the surface of EVs. While GPC1 was unable to discern pancreatic cancer from BPD, nFC technology opens up the possibility of the re-examining previously-identified pancreatic cancer markers in novel combinations to develop an effective early detection test for pancreatic cancer.

## MATERIALS AND METHODS

### Plasma samples, patient characteristics and reagents

93 de-identified patient plasma samples were obtained from the Princess Margaret Cancer Centre in Toronto, ON, Canada (Dr. Steve Gallinger, University of Toronto) (Table 1). 41 plasmas representing negative control samples were collected from patients with benign pancreatic diseases such as pancreatitis and pancreatic cysts/pseudocysts that were false positives through the CA 19-9 serum test for pancreatic cancer. Clinical follow-up of this cohort is in [30]. All plasma samples were collected with permission from the University of Western Ontario Research Ethics Board and under protocol ID 103603. 52 samples were obtained from patients with histologically-confirmed exocrine pancreatic cancer, 22 samples were obtained from patients with resected pancreatic cancer (who were subsequently assessed to have stage IA-IIB cancer), and 30 samples were obtained from patients with metastatic (stage IV) pancreatic cancer. All plasmas were collected under informed patient consent from all patients and if the subject was under 18 y.o., from a patient and/or legal guardian. Plasmas were stored at -80°C until use. All methods were carried out in accordance with relevant guidelines and laboratory safety protocols and regulations. All experimental protocols were approved by Lawson Health Research Institute and the University of Western Ontario.

Rabbit polyclonal GPC1 antibody, PA5-24972 (Thermo Fisher Scientific Inc.) was directly conjugated to Alexa Fluor 647 dye using the Zenon Alexa Fluor 647 Rabbit IgG Labelling Kit, Z-25002 (Thermo Fisher Scientific Inc.). Similarly, mouse monoclonal GP2 antibody (NCI Hybridoma GP2.2863, Deeley Research Centre, purified by AbLab, University of British Columbia) was directly conjugated to Alexa Fluor 488 dye using the Zenon Alexa Fluor 488 Mouse IgG1 Labelling Kit, Z-25308 (Thermo Scientific). Conjugation of the antibodies using the Zenon labeling kits was performed according to the manufacturer's suggested protocol for the use of antibodies in flow cytometry.

### Nanoscale flow cytometry analysis

Nanoscale flow cytometry analysis of patient plasma samples was performed in a blinded manner. One microliter of GPC1-Alexa Fluor 647 [50 ng/μL] and GP2-Alexa Fluor 488 [100 ng/μL] pre-conjugated mAb was added to 20 μL of patient plasma and the sample was incubated in the dark for 20 minutes at room temperature. PBS was added to the plasma samples to achieve a total volume of 600μL (30-fold dilution) and samples were subsequently analyzed via the A50-Micro Nanoscale Flow Cytometer (Apogee FlowSystems Inc.) for GPC1-positive, GP2-positive, and GPC1+GP2-positive EVs between 100-1,000nm in diameter. For each patient, we also incubated plasma samples with pre-conjugated rabbit IgG-Alexa Fluor 647 isotype control (bs-0295P-A647, Bioss Inc.) and mouse IgG1-Alexa Fluor 488 isotype control (ab171463, Abcam Inc.) following the same protocol used for nanoscale flow cytometry analysis for GPC1 and GP2 as outlined above. Positive counts in the isotype controls were subtracted from the counts observed in corresponding samples incubated with GPC1 and GP2 antibodies in order to correct for the level of non-specific binding.

Prior to sample analysis, calibration of the nanoscale flow cytometer was performed using a reference bead mix (*ApogeeMix*, Apogee Flow Systems Inc.) composed of a mixture of silica nanoparticles with diameters of 180 nm, 240 nm, 300 nm, 590 nm, 880 nm, and 1,300 nm with a refractive index (RI) of 1.42; and 110 nm and 500 nm green fluorescent (excited by blue laser) polystyrene nanoparticles with an RI of 1.59 (latex) were used. These beads were used to assess the nanoscale flow cytometer's (nFC) light scattering detection of extracellular vesicles (microvesicles, microparticles, exosomes etc.) and fluorescence detection resolution. Thresholds and PMTs were set to eliminate optical and electronic background noise without losing particles of interest.

### Immunohistochemistry of Tissue Microarrays

The pancreatic cancer SPORE Tissue microarray (TMA) consists of 342 cores representing 140 different individuals with pancreatic ductal adenocarcinoma (PDAC) and placed on the TMA using a random layout. Immunohistochemistry was performed with GPC1 antibody (PA5-24972, Thermo Fisher Scientific Inc.) and tumors were scored with Aperio ImageScope (Leica Biosystems Inc.). Cytoplasmic/membrane staining was quantified with the Aperio Cytoplasm Agorithm. Paraffin sections of breast cancer and pancreatic cancer xenografts were used as positive controls for GPC1 staining. GPC1-positive breast cancer cells (MDA-MB 231) and pancreatic cancer cells (PANC-1) were onplanted onto chorioallantoic membrane of chicken embryos [31, 32]. After 7 days of growth *in vivo*, tumors formed were extracted and embedded in paraffin or OCT for immunohistochemistry analysis.

### Statistical analysis

Nanoscale flow cytometry data was collected using A50-Micro instrument acquisition software. Data was analyzed using GraphPad Prism version 6.0 (GraphPad Software). A one-way ANOVA test was used to evaluate any statistical significance between the GPC1-positive and GPC1-GP2-positive EV counts observed in the different patient groups. ROC curves were used to determine the area under the curve (AUC), sensitivity, specificity, and cut-off values for the GPC1 and GPC1-GP2 tests in order to characterize their effectiveness in the diagnosis of pancreatic cancer. For the generation of ROC curves, we considered the positive outcome to be metastatic pancreatic cancer and the negative outcome to be BPD (patient data for the resected pancreatic cancer group were not used for the generation of ROC curves).

## SUPPLEMENTARY MATERIALS FIGURES AND TABLES


