# Supplementary material for: Glypican-1 and glycoprotein 2 bearing extracellular vesicles do not discern pancreatic cancer from benign pancreatic diseases
Source: Oncotarget. 2019 Feb 1;10(10):1045–55. doi: 10.18632/oncotarget.26620 (PMC6383691; doi:10.18632/oncotarget.26620)
Supplement: Supplementary file 1 [file oncotarget-10-1045-s001.pdf]

Glypican-1 and glycoprotein 2 bearing extracellular vesicles do not discern pancreatic cancer from benign pancreatic diseases

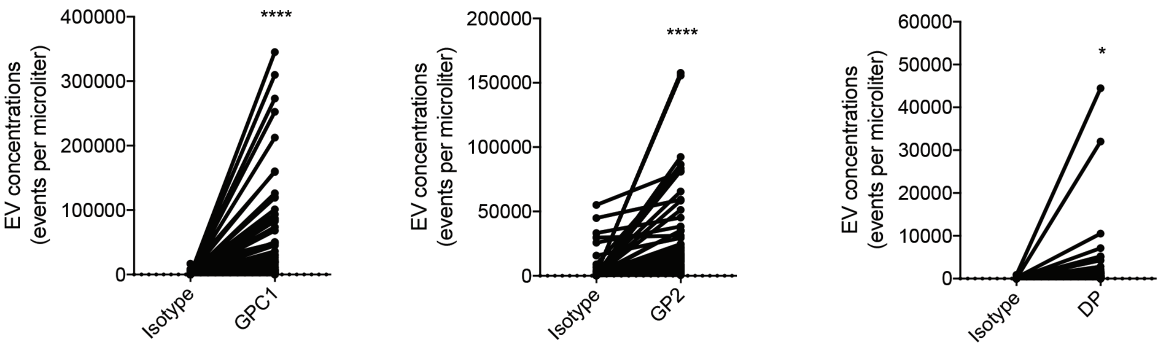

	Isotype	GPC1
Number of values	93	93
Minimum	39.3	240
25% Percentile	220	1806
Median	677	7121
75% Percentile	1210	40700
Maximum	16839	345160

Two-tailed Paired t-test
p<0.0001

	Isotype	GP2
Minimum	240	600
25% Percentile	580	2912
Median	840	6760
75% Percentile	1300	19840
Maximum	55080	157731

Two-tailed Paired t-test
p<0.0001

	Isotype	DP
Minimum	0	0
25% Percentile	0	40
Median	0	120
75% Percentile	0	440
Maximum	866	44459

Two-tailed Paired t-test
p=0.016

Supplementary Figure 1: Nanoscale flow cytometric evaluation and results of isotype stained plasma samples. Corresponding isotype values are listed for each patient plasma sample analyzed. Each table lists the results generated for each biomarker-isotype pair.

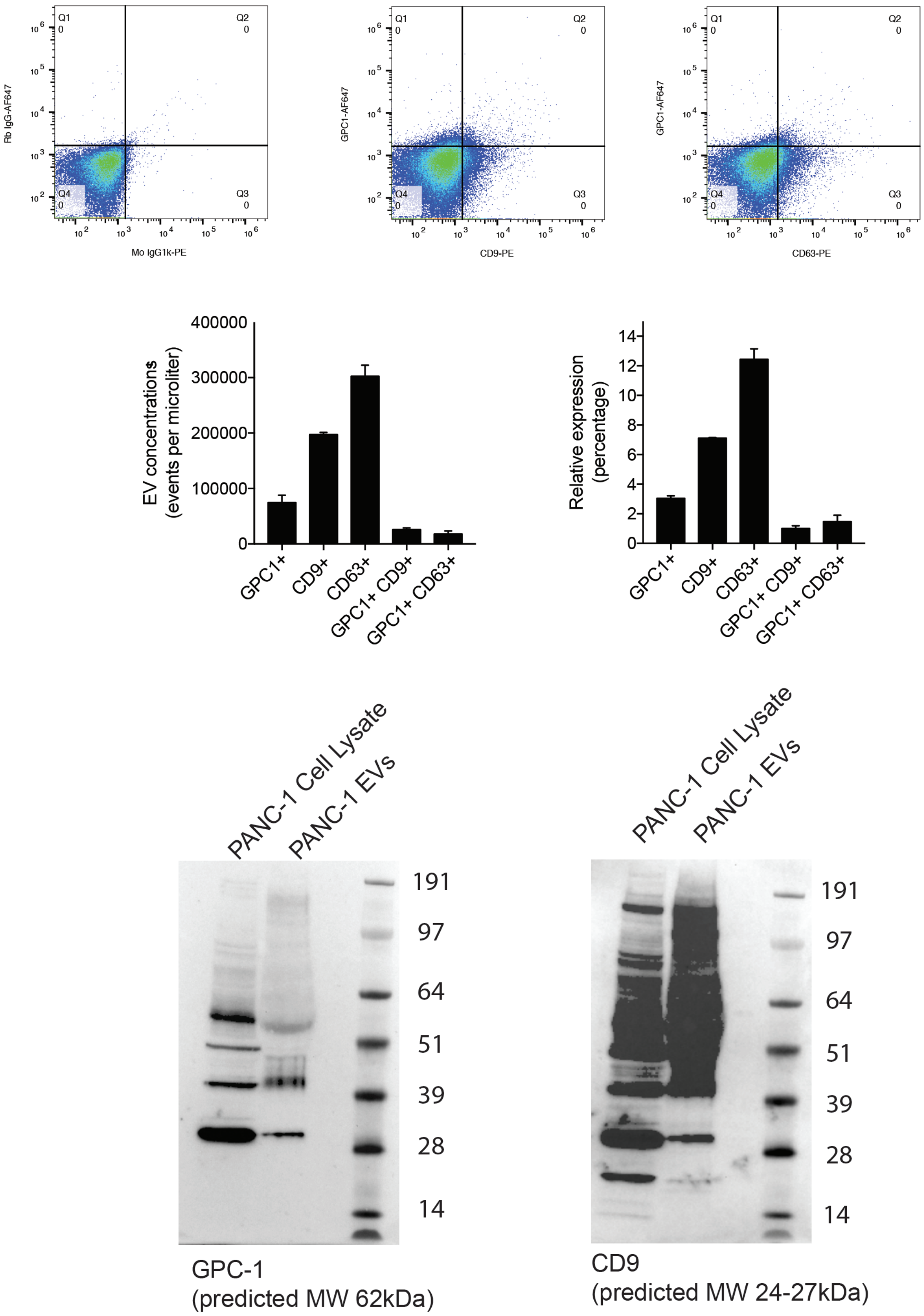

Supplementary Figure 2: Nanoscale flow cytometry (top and middle panels) and Western Immunoblot analysis of PANC-1 cells. GPC1 and CD9 expresion on EVs released by PANC-1 cells was observed and quantitated (top and middle panels). Western immunoblot analysis confirmed the true EV origin of GPC1-positive EVs. As expected, GPC1- and CD9-positive EVs are detected by both techniques. The presence of GPC1+ CD9- EVs and GPC1- CD9+ EVs in PANC-1 culture media was also observed (bottom panel).
